# Development of learning objectives for neurology in a veterinary curriculum: part I: undergraduates

**DOI:** 10.1186/s12917-014-0315-3

**Published:** 2015-01-13

**Authors:** Yu-Wei Lin, Holger A Volk, Jacques Penderis, Andrea Tipold, Jan P Ehlers

**Affiliations:** Department of Small Animal Medicine and Surgery, University of Veterinary Medicine Hannover, Hannover, Germany; Department of Clinical Science and Services, Royal Veterinary College, University of London, London, UK; Department of Clinical Neurology, School of Veterinary Medicine, University of Glasgow, Glasgow, Scotland; Didactics and Educational Research in Health Sciences, Witten-Herdecke University, Witten-Herdecke, Germany

**Keywords:** Veterinary education, Curriculum, Learning objectives, Neurology, Undergraduate, ECVN, ESVN, Europe

## Abstract

**Background:**

With an increasing caseload of veterinary neurology patients in first opinion practice, there is a requirement to establish relevant learning objectives for veterinary neurology encompassing knowledge, skills and attitudes for veterinary undergraduate students in Europe. With help of experts in veterinary neurology from the European College of Veterinary Neurology (ECVN) and the European Society of Veterinary Neurology (ESVN) a survey of veterinary neurologic learning objectives using a modified Delphi method was conducted. The first phase comprised the development of a draft job description and learning objectives by a working group established by the ECVN. In the second phase, a quantitative questionnaire (multiple choice, Likert scale and free text) covering 140 learning objectives and subdivided into 8 categories was sent to 341 ESVN and ECVN members and a return rate of 62% (n = 213/341) was achieved.

**Results:**

Of these 140 learning objectives ECVN Diplomates and ESVN members considered 42 (30%) objectives as not necessary for standard clinical veterinary neurology training, 94 (67%) were graded to be learned at a beginner level and 4 (3%) at an advanced level. The following objectives were interpreted as the most important day one skills: interpret laboratory tests, perform a neurological examination and establish a neuroanatomical localization. In this survey the three most important diseases of the central nervous system included epilepsy, intervertebral disc disease and inflammatory diseases. The three most important diseases of the peripheral nervous system included polyradiculoneuritis, myasthenia gravis and toxic neuropathies.

**Conclusions:**

The results of this study should help to reform the veterinary curriculum regarding neurology and may reduce the phenomenon of “Neurophobia”.

**Electronic supplementary material:**

The online version of this article (doi:10.1186/s12917-014-0315-3) contains supplementary material, which is available to authorized users.

## Background

In the 1950s Bloom published “*Taxonomy of educational objectives: the classification of educational goals*” [1,2], which established learning objectives as one of the most important concepts in pedagogy. By clearly defining learning objectives, the assessment and evaluation become independent from the instructional mode used or the subjective opinions of the teachers [3]. Learning objectives are the educational foundation of a competence-oriented curriculum, which indicate the expectation of teaching/learning and the assessment thereof. The learning objectives define (A) WHO can (B) DO (C) WHAT (D) HOW MUCH or HOW WELL [4]. These abbreviations are symbols for an (A) AGENT (in this case a specifically addressed learner), who’s specific (B) ACTION will be executed by a defined (D) PERFORMANCE LEVEL, in order to prove his learned knowledge, abilities or behavior of a given (C) CONTENT [4]. In other words, learning objectives define specifically what knowledge, skills and attitudes learners should obtain. These should be “SMART”:

- **S**pecific
- **M**easurable / Observable
- **A**ttainable for target audience within scheduled time and specified conditions
- **R**elevant and results-oriented
- **T**argeted to the learner and to the desired level of learning [5].

In a medical school setting, the professional training “would be extremely inefficient without a blueprint of knowledge, skills and attitudes transmitted by instructors and acquired by students. Without such a plan, a tight overlap between what is being taught, learned and examined could not be guaranteed” [6], R. Bloch expressed here the importance and the necessity of involvement of learning objectives being the core of any good curriculum. Essential objectives help undergraduates to gain confidence and to focus on their learning process.

In veterinary medicine, as is the case in human medicine, neurology is recognized as an separate specialty [7]. During the 1990s - “the Decade of the Brain”, neurological disorders were given national attention in the United States [8]. Due to the increasing life expectancy of people it was predicted that neurologic problems would become increasingly important within the human population [9]. To ensure the quality of neurologic training for all physicians, a process to define the core curriculum for neurology was initiated in October 1998, under the auspices of the Consortium of Neurology Clerkship Directors (CNCD) and the Undergraduate Education Subcommittee (UES) of the American Academy of Neurology (AAN) [10].

Veterinary neurology is a flourishing specialization in Europe and the United States. Neurological diseases are common in veterinary practice and the level of understanding of these conditions has dramatically increased over the past few decades [11]. A search of the Web of Knowledge using the following parameters: “Topic = (dog) OR Title = (cat) AND Topic = (neuro)” reveals a significant increase of published items over the last 15 years, with almost 9000 publications in total. The growth of the veterinary neurology has also resulted in increased expectations from pet owners for their animals to receive specialized care [12]. A recent study from the Royal Veterinary College found that the cause of death in dogs in the United Kingdom was due to neurological cranial disease (including seizures) in 8,38% of dogs [13], which reflects the requirement for neurology training in veterinary medicine. Consequently, undergraduate students need to be taught the basic principles of the discipline, must be able to recognize the clinical signs of neurologic disease, be able to manage neurologic emergencies, and know when to refer cases to specialists or have the necessary skill base to allow them to start a specialist training themselves.

Although learning objectives for veterinary neurology have been defined in many individual universities, Europe wide detailed learning objectives for veterinary neurology as well as other subjects have not yet been defined. There is a requirement for such objectives for veterinary neurology to be established in Europe. This could be achieved in a similar manner to the development of the US undergraduate curriculum, which was developed using the expert opinions of CNCD and AAN, with the help of certified and recognized specialists in Veterinary Neurology (e.g. European Diplomates of the European College of Veterinary Neurology (ECVN)) and advanced practitioners with a special interest in Veterinary Neurology (European Society of Veterinary Neurology (ESVN) members). The designated learning objectives could be used to define the basic necessary knowledge, skills and attitudes for undergraduate students in veterinary neurology. Assembled in the curriculum they would form the basis for competency-based training and outcome-based assessment and could motivate undergraduates towards postgraduate specialist training in the discipline.

The aim of the current study was to develop learning objectives for undergraduates using information gained via a survey of ECVN and ESVN members. The “undergraduate” in the study was referred to the pre-clinical and clinical year; moreover, the participants of the survey were informed to judge the level that undergraduate should reach after their clinical year. The international profile of the members helped to create learning objectives largely independent of cultural background. Experts helped to keep the contents of learning objectives “as much knowledge as necessary” and “as little knowledge as possible”. In addition to the development of the learning objectives, the quality and level of these were defined. The current approach of curriculum development is not specific for Neurology and could also be used for other disciplines.

This study contains only part of the results of the whole survey. The participants of the survey were asked to judge each learning objectives for three groups: 1. for undergraduates, 2. for advanced practitioners and 3. for residents and ECVN Diplomates. The part including learning objectives for Residents and required job competencies of Diplomates of the European College of Veterinary Neurology will be published in part II.

## Methods

A modified Delphi method was conducted to identify relevant learning objectives. Draft learning objectives were developed with the help of an ECVN curriculum-working group, the revised learning objectives were then assessed by Experts (ECVN and ESVN members) and the responses of these Experts were statistically analyzed.

### Phase 1

Qualitative development of a draft of learning objectives with the help of an ECVN curriculum working group.

The draft was based on the structure and learning objectives recently created by the American College of Veterinary Internal Medicine (ACVIM) for evaluating the competencies of their residents (postgraduate veterinarians in a formal training program) in Neurology. The draft of learning objectives was reviewed and adapted by the ECVN curriculum working group, this group consisted of seven ECVN Diplomates* from different Universities and private practices in Europe. Following the review of the draft the initial list of learning objectives were comprehensively revised.

### Phase 2

A quantitative questionnaire with revised learning objectives was distributed to ESVN members (comprising 142 veterinarians specially interested in neurology and 72 ECVN residents) and 127 ECVN members (Diplomates of the ECVN).

In phase 1, a total of 140 learning objectives (Additional file 1) in 8 categories were developed (1. Anatomy and Physiology; 2. Pharmacology and Toxicology; 3. Genetics and Molecular Biology; 4. Clinical Methodology; 5. Disease Mechanisms; 6. Neuroanesthesia and Neurosurgery; 7. Neuroradiology; 8. Pathology). In the category Clinical Methodology, the abilities of performance and interpretation were assessed, in Neuroanesthesia/Neurosurgery and Neuroradiology the learning objectives were sub-categorized into theory and practice.

The developed quantitative questionnaire with all these learning objectives was then distributed to 341 ESVN and ECVN members using Surveymonkey® (an online-survey provider). Every member received a unique link by e-mail for the questionnaire, which was active for 3 months. The users could pause and continue the questionnaire at any time during the active period. All data of this study were used anonymously and treated confidentially according to the EU Data Protection Directive 95/46/EC. The clearance for this research project was given by the data protection officer of the University of Veterinary Medicine Hannover and followed the ethical regulations of the university.

The questionnaire was compounded of single/multiple choice questions for demographic data, Likert scale for learning objectives and free text for comments. Respondents were requested to indicate the importance of the learning objectives for undergraduates based on Bloom’s taxonomic classification [14,15] using the following Likert scale:

- 1 = Not Necessary
- 2 = As Beginner - Theory knowledge: knowing terms
- Practice Skills: knowledge of theory by practice
- 3 = As Advanced - Theory: Being able to interpret
- Practice Skills: perform under instruction by practice
- 4 = As Expert - Theory: Being able to discuss intellectually
- Practice Skills: perform independently

Additionally, an option “No Idea” was available, and responses of this option were excluded from statistic analysis.

### Phase 3

Statistical evaluation using Fisher’s Exact Test.

To see if there were biases influencing the results of the survey several groups of respondents were created and compared to each other. All questions used the same Likert scale, which made the scale a defensible approximation to an interval scale. After consulting the statistical support service of the Institute of Biometrics of the University of Veterinary Medicine Hannover, the non-parametric Fisher’s Exact Test was used with statistic software SAS® Version 9.2 under the assumption of unequal variances, two-tailed distributions and a significance level of 0.05. In addition, the responses were evaluated among the following groups to discover different opinions:

1. ESVN vs. ECVN
2. German-speaking vs. non-German-speaking countries
3. Surgery vs. no-surgery performed
4. Experience in Neurology: 0–5 Years vs. 6–10 Years vs. > 10 Years.

The free text answers were also summarized and qualitatively presented, the three most important objectives would be presented. An overall view of all learning objectives with mean values and level distribution was attached as Additional file 1.

*Members of ECVN curriculum working group included H.A. Volk, J. Penderis, T. Anderson, S. Añor, A. Lujan-Feliu-Pascual, V.M. Stein and A. Tipold.

## Results

The questionnaire was sent to 341 experts with a overall response rate of 62% (n = 213/341), of which 77% (164/213) submitted a completed questionnaire and were included in the analysis.

The completed questionnaires were from 83 ESVN (including 46 residents) and 81 ECVN-Diplomate members. The majority of the respondents worked in the United Kingdom (44), Germany (30), Italy (23) and Spain (15). 45% of the respondents worked in academia, 44% in private specialty practice, 8% in both areas and 3% in industry or other organizations. Furthermore, 97% of the respondents worked mainly with small animals.

Of 140 learning objectives, 42 (30%) learning objectives were considered as not necessary for undergraduates, 94 (67%) were considered required to be achieved at beginners level, 4 (3%) at advanced level and none at expert level (Additional file 1). The 42 disregarded objectives were in the area of electrodiagnostic tests (57%; n = 24/42), performing CSF puncture, most surgical techniques and advanced techniques in neuroradiology (Additional file 1).

The ten learning objectives with the highest mean rating (2.58-2.25, beginner to advanced level) are listed below (Table 1). They could be considered as “day one skills” for undergraduates in neurology. The first five learning objectives did not include specific neurologic themes, but were transferable skills necessary for accurate neurologic diagnoses, and the last five were associated with neuroanatomical localization, general clinical reasoning and with specific common disease presentations (intervertebral disc disease and seizures). A list of day one skills containing only neurologic competencies was listed in Table 2.

**Table 1:** **Rating of 10 most important learning objectives**

**Learning objectives**	**Mean rating**
- Interpret hematological, serum chemistry and urinalysis results	2.58
- Understand organ function tests (liver, endocrine).	2.58
- Interpret organ function tests (liver, endocrine).	2.56
- Interpret radiographs of the abdomen and thorax.	2.50
- Interpret radiographs of the axial and appendicular skeleton.	2.41
- Neurolocalize a lesion based on the examination findings.	2.37
- Understand CNS diseases according to the VITAMIN-D principal	2.37
- Understand the diagnosis and treatment of disc disease in dogs and cats.	2.35
- Understand the pathogenesis of disc disease in dogs and cats.	2.32
- Understand the diagnosis and treatment of seizure in dogs and cats	2.29

**Table 2:** **Rating of 10 most important “neurologic” learning objectives**

**Learning objectives**	**Mean rating**
- Neurolocalize a lesion based on the examination findings.	2.37
- Understand CNS diseases according to the VITAMIN-D principal	2.37
- Understand the diagnosis and treatment of disc disease in dogs and cats.	2.35
- Understand the pathogenesis of disc disease in dogs and cats.	2.32
- Understand the diagnosis and treatment of seizure in dogs and cats	2.29
- Perform a neurologic examination of all species	2.24
- The side-effect profiles of the immunosuppressive drugs for CNS inflammatory disease	2.21
- The gross neuroanatomic structures of the cat and dog brain and spinal cord	2.19
- The mechanism of action of pain therapy	2.19
- Ability to interpret radiographs of the skull	2.18

In free text questions the respondents named the three most important antiepileptic drugs currently used in veterinary neurology and these included benzodiazepine, phenobarbital, potassium bromide (following levetiracetam, gabapentin and zonisamide); the three most important immunosuppressive or anti-inflammatory drugs were glucocorticosteroids, azathioprine and cyclosporine; the three most important chemotherapeutic drugs groups were: nitrosoureas, cytosine arabinoside and nitrogen mustards.

The three most important diseases of the central nervous system (CNS) that respondents thought that an undergraduate veterinary student should be knowledgeable about included epilepsy, intervertebral disc disease and inflammatory diseases of CNS. The three most important diseases of the peripheral nervous system (PNS) were considered to be polyradiculoneuritis, myasthenia gravis, neurotoxins.

### Evaluation of the learning objectives by ESVN or ECVN members

**Figure 1:**
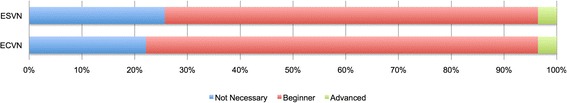
**Distribution of expected level from the groups ESVN and ECVN.** By ESVN, 26% (n = 36) of learning objectives would be considered as not necessary, 71% (n = 99) as beginner and 3 (n = 5) as adcanced. By ECVN, 22% (n = 31) as not necessary, 74% (n = 104) as beginner and 4% (n = 5) as advanced.

All 164 completed questionnaires were included in the analysis (83 ESVN members and 81 ECVN-Diplomates). Interestingly, there was no difference between ESVN and ECVN members in what level they expected from an undergraduate veterinary student (Figure 1). Of the 140 learning objectives, significant differences (P < 0.05) were detected in only 8 learning objectives (Table 3), of which 6 learning objectives received a higher rating from ECVN Diplomates.

**Table 3:** **Rating of the importance of learning objectives: comparison between the ECVN and ESVN group; 8 learning objectives were rated significantly different**

	**Mean ECVN**	**Mean ESVN**	**P-value**
**Anatomy and Physiology**			
Understand the microscopic anatomy of the nervous system	**1.82**	1.56	0.0145
Understand the functional neuroanatomy of the central nervous system	**2.25**	2.04	0.0124
Understand the functional neuroanatomy of the autonomic nervous system	**2.09**	1.89	0.0027
**Clinical Methodology**			
**> Laboratory**			
Interpret hematological, serum chemistry and urinalysis results	**2.63**	2.52	0.035
**> CSF**			
Perform cistern magna collection of CSF in the dog and cat	**1.55**	1.32	0.0398
**> EMG**			
Interpret EMG and nerve conduction testing in the dog and cat.	1.38	**1.63**	0.005
**Neuroradiology**			
**> Practical**			
Interpret radiographs of the skull	2.08	**2.28**	0.0328
**Pathology**			
Understand hematological cytological interpretation	**2.38**	2.13	0.027

Values in boldface have a higher mean rating.

### Evaluation of the learning objectives by experts who work in German-speaking or in non-German-speaking countries

**Figure 2:**
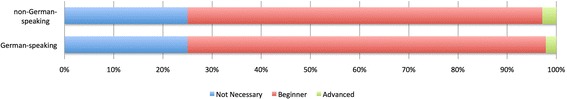
**Distribution of expected level: comparison between who work in German-speaking and non-German-speaking countries.** By German-speaking, 25% (n = 35) of learning objectives would be considered as not necessary, 73% (n = 102) as beginner and 2% (n = 3) as advanced. By non-German-speaking, 25% (n = 35) as not necessary, 72% (n = 101) as beginner and 3% (n = 4) as advanced.

38 Respondents were working in German-speaking countries, 126 respondents in non-German-speaking countries. In this comparison, the expectation from both groups was almost identical (Figure 2). Only 3 learning objectives were graded significantly different (Table 4).

**Table 4:** **Rating of the importance of learning objectives: comparison between respondents who work in German-speaking and non-German-speaking countries**

	**Mean rating German-speaking**	**Mean rating Non-German-Sp.**	**P-value**
**Anatomy and Physiology**			
Understand the functional neuroanatomy of the peripheral nervous system	2,05	**2,14**	0,0278
**Pharmacology and Toxicology**			
Understand the therapeutic index in relation to drug efficacy and safety	**2,08**	1,93	0,0431
**Neuroradiology**			
Understand CT scanning technique	**1.84**	1,78	0,0381

Values in boldface have a higher mean rating.

Three 3 learning objectives were rated significantly differently.

### Evaluation of the learning objectives by experts, who do perform or do not perform neurosurgery

**Figure 3:**
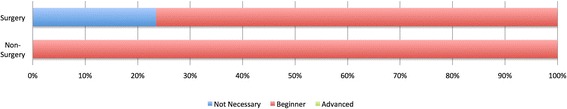
**Distribution of expected level from the groups “offering surgery” and “not-offering surgery”.** By “offering-surgery”, 24% (n = 4) of learning objectives would be considered as not necessary, 76% (n = 13) as beginner and none of them as advanced. By “non-perform-surgery”, none as not necessary, 100% (n = 17) as beginner and none as advanced.

This evaluation was only performed in the category neuroanesthesia/neurosurgery with 17 (4 theoretical and 13 practical skills) learning objectives. The group of respondents not performing surgery expected all 17 learning objectives to reach beginner’s level; in contrast, respondents performing-surgery rated 4 skills as not necessary (Figure 3). Though no significant difference was detected between the two groups, respondents not performing surgery had higher expectations.

### Evaluation of the learning objectives by Experts, who have experience in veterinary neurology for 0–5, 6–10 or >10 Years

**Figure 4:**
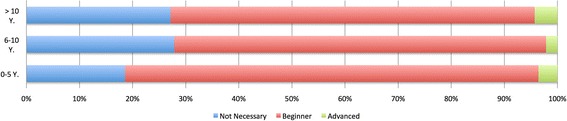
**Distribution of expected level from the groups with different experience in neurology (0–5, 6–10 and >10 years).** By experience with 0–5 years, 19% (n = 26) of learning objectives would be considered as not necessary, 78% (n = 109) as beginner and 3% (n = 5) as advanced. By 6–10 years, 28% (n = 39) as not necessary, 70% (n = 98) as beginner and 2% (n = 3) as advanced. By >10 years, 27% (n = 38) as not necessary, 69% (n = 96) as beginner and 4% (n = 6) as advanced.

When comparing groups of different experience levels, the members of the 0–5 years’ group expected more learning objectives to reach beginner’s level than the group with 6–10 years of experience or >10 years (Figure 4). Significant differences were detected between the groups (Table 5). Moreover, the group with 0–5 years experience expected 6 learning objectives of 24 in the category of electrodiagnostics to reach beginner’s level, while the other 2 groups regarded all as not necessary.

**Table 5:** **Competencies with significant difference between the groups with different experience in neurology (0–5, 6–10, >10 years)**

	**Mean 0-5**	**Mean 6-10**	**Mean >10**	**P-values**
**Anatomy and Physiology**				
Understand the microscopic anatomy of the nervous system	1.55***	1.69	1.8***	0.031***
Understand the functional neuroanatomy of the autonomic nervous system	1.87***	1.9	2.17***	0.0326***
**Pharmacology and Toxicology**				
**> pharmacodynamic and Pharmacokinetic**				
Understand the autonomic nervous system receptors and neurotransmitters	1.72***	1.81	1.97***	0.04***
**> Chemotherapeutic drugs**				
Understand the mechanism of chemotherapeutic drugs for nervous system neoplasia/inflammation	1.69	1.65**	1.88**	0.028**
**Clinical Methodology**				
**> EEG**				
Perform EEG testing in the dog and cat	1.43* ***	1.16*	1.19***	0.0371* 0.0121***
Interpret EEG testing in the dog and cat	1.48* ***	1.17*	1.22***	0.0273* 0.0124***
**> EMG**				
Perform EMG and nerve conduction testing in the dog and cat	1.48*	1.19*	1.25	0.0329*
Perform F-waves, Repetitive stimulation and H-wave testing in the dog and cat.	1.44* ***	1.13*	1.16***	0.0254* 0.0066***
Interpret F-waves, Repetitive stimulation and H-wave testing in the dog and cat.	1.56* ***	1.2*	1.23***	0.0154* 0.0033***
Interpret EMG and nerve conduction testing in the horse.	1.53* ***	1.28*	1.27***	0.0207* 0.0062***
Interpret single fiber EMG testing in the dog and cat.	1.31*	1.1*	1.17	0.0207*
**> OPHTAMOLOGIC ELECTRO. TESTING**				
Perform ophthalmologic electrodiagnostic testing (ERG, VEP) in the dog and cat.	1.46* ***	1.1*	1.13***	0.0155* 0.0036***
**Disease Mechanisms**				
**> Micturition Disorders**				
Understand the pathogenesis of micturition disorders of dogs and cats	2.19*	1.95*	2.1	0.0377*
Understand the pathogenesis of micturition disorders of horses	1.82***	1.68	1.56***	0.0156***
Understand the diagnosis and treatment of micturition disorders of horses	1.88***	1.72	1.56***	0.0263***
Understand the pathogenesis of micturition disorders of ruminants/food animals	1.79***	1.54	1.49***	0.0336***
Understand the diagnosis and treatment of micturition disorders of ruminants/food animals	1.85***	1.57	1.48***	0.0073***
**> Seizure**				
Understand the pathogenesis of seizure disorders in horses	1.9***	1.74	1.75***	0.0255
Understand the pathogenesis of seizure disorders in ruminants/food animals	1.75***	1.91**	1.64** ***	0.003** 0.0389***
**> Disc Disease**				
Understand the pathogenesis of disc disease in dogs and cats	2.31	2.17**	2.44**	0.0487**
**Neuroanaesthesia & Neurosurgery**				
**> Practical**				
Perform Brain biopsy	1.69* ***	1.38*	1.36***	0.0252* 0.0125***
Perform Fracture repair	1.7*	1.4*	1.48	0.0223*
Perform Muscle biopsy	1.79*	1.6*	1.6	0.0298*
Perform nerve biopsy	1.64* ***	1.48*	1.49***	0.012* 0.0111***
**Neuroradiology**				
Understand CT scanning technique	1.85***	1.69**	1.84** ***	0.0415** 0.0108***
Understand MRI scanning technique	1.75	1.57**	1.79**	0.0283**

Values with *, indicate the significance of the learning objectives between groups 0-5 and 6-10; for groups 6-10 and >10 are indicated with **; and groups 0-5 and >10 with ***.

## Discussion

The goal of this study was to determine a catalog of learning objectives for veterinary neurology undergraduate curricula in a European framework. The statistical results of the returned questionnaires show interesting findings between different groups. Experts from areas of teaching, research and practice were involved in the first phase to develop a draft of learning objectives.

97% of the respondents worked mainly with small animals, reflecting the main working area of employment of veterinary neurologists. Further examinations, such as electroencephalography, myelography, computed tomography or magnetic resonance imaging are mostly performed as routine tools in small animals and only to a smaller extent in large animals.

Only for 4 (3%) of the 140 learning objectives the ESVN/ECVN group felt undergraduates should reach an advanced level. These objectives were all listed in the categories laboratory and radiology and were non-neurology specific. The undergraduates should be able to understand and interpret the result of hematology, serum chemistry, urinalysis and organ function test and radiographs of the abdomen and thorax. The ten learning objectives (Table 1) with the highest mean rating could be considered as the neurology day one skills for undergraduates, which also include five general transferable skills.

Undergraduates were expected to reach beginner level of understanding (knowing terms by theory or knowledge and comprehension of theory by practice) for 67% (94/140) of the analyzed learning objectives. These objectives would be ranked relatively low in the cognitive domain of Bloom’s Taxonomy. In addition, 30% (42/140) of the learning objectives in the categories of electrodiagnostic tests, CSF puncture, bone marrow aspiration, biopsy, advanced neurosurgical skills and neuroradiological techniques were considered as not necessary for undergraduate students. Based on our findings undergraduates should have basic understanding of most of the analyzed objectives, however, they should be motivated to further their knowledge and skill sets.

To see if the results were influenced by the role of the respondents (interested practitioners vs. an exclusive specialization) the members of ECVN and ESVN were compared separately. They ranked all learning objectives similarly. ECVN members gave, however, higher mean ratings than ESVN members in eight of the learning objectives. The different working environment may explain this phenomenon; part of the ESVN member group are veterinarians who are especially interested in neurology, however, neurology cases are not their primary and only caseload. In contrast, ECVN Diplomates are mainly working in academia, university hospitals or specialist referral clinics and therefore their routine caseload is neurology based. In this study, 46 residents participated in the study. Results were included in the ESVN group. Residents are a heterogenous group concerning knowledge, considered to be trainees and only to a smaller degree as trainers in comparison to ECVN Diplomates. Other ESVN members might have a similar trainer status as residents, when they are responsible for extramural training of students.

In order to evaluate if a defined group of European countries had different opinions from those of other countries and to examine if there was a bias because the study and survey were organized from German researchers, German-speaking countries were evaluated separately and compared with the others. There were only three learning objectives that demonstrated significant differences between the two groups. The international community of the ESVN and ECVN, in particular their regular meetings, may contribute to this uniform result. Furthermore, the mission of the EAEVE (European Association of Establishment for Veterinary Education) is to ensure a comparable quality of veterinary medical education across the member states of the European Union [16], which may also be an explanation for this phenomenon.

The respondents were divided in groups performing or not perfoming neuro-surgery to see if this former specialization influences the answers to the survey. Experts who performed surgery agreed that four of the seventeen objectives in the category neuroanesthesia/neurosurgery were not necessary. On the other hand experts who didn’t perform surgery expected all learning objectives to reach beginner’s level (knowing terms or knowledge of theory by practice). Even if this difference was not significant, it shows a tendency for experts in surgery to have less high expectations than medical neurologists. Neurosurgery is a specific area in surgery. For undergraduates an advanced or expert level should not be considered necessary. However, they should know the terms by theory and understand the knowledge of theory by practice via for example lectures, seminars, eLearning or skills laboratories. Miller describes the assessment of clinical skills as a pyramid and suggests that the undergraduate student should reach the second level “Know How”, which means the undergraduate should “Know” and/or “Know How” a certain clinical procedure is performed, but it is not yet necessary to reach the “Show How” level [17]. Fundamental knowledge for surgery can be acquired passively by lectures and the active learning usually takes place during clinical rotations [18]. In skills laboratories various simulators provide hands-on training, representing alternative possibilities for different psychomotor objectives [19].

The interesting and surprising finding in this comparison was that the less experienced group expected more learning objectives for undergraduates to reach a beginner’s level and also gave higher mean ratings than the groups with more experience. In addition, less experienced group also demonstrated greater interest in electrodiagnostic tests and considered them more important than another groups. In the 24 learning objectives of electrodiagnostic tests the less experienced group expected undergraduates to reach beginner level in 6 (25%) learning objectives, while the 2 other groups regarded all of them as unnecessary.

Because veterinary neurology is very closely associated with a number of different veterinary disciplines, implementation of the learning objectives in the current curricula with an increasing interdisciplinary cooperation would be preferable. With the help of inter-institutional support and expertise from different fields of veterinary science, a meaningful interdisciplinary cooperation provides valuable teaching and learning synergies [20].

Additionally, elective courses could also be offered. Moreover, E-learning is an ideal supplement to classroom education. An example is the platform CASUS® providing various interactive neurology themes for veterinary undergraduates, which is regarded as an efficient teaching method [21,22]. Using such tools the most important diseases can be provided for self-study. A study performed in the UK describes the phenomenon “Neurophobia” around human medical students [23]. Such undergraduates may profit using different media to help learning the broad field of neurology. Moreover, Ridsdale et al. mentioned also the phenomenon of neurophobia in human medicine and suggested that one of the reasons of neurophobia may comes from unfocussed neurology teaching [24], because knowledge of neurology are often taught in different parts of discipline. A complete and transparent list of learning objectives therefore may help trainers by curriculum designing and the undergraduates will profit from it as well. The undergraduates can concentrate on essential knowledges and the transparency of these learning objectives may help them reduce the neurophobia.

With the result of this pilot study, we expect that veterinary neurology, as a niche discipline, would not only provide the orientation for training of undergraduates in veterinary neurology, but might also be a role model for the development of European learning objectives in other specific areas in veterinary medicine.

One of limitations of this study is that some of the learning objectives listed in the survey were not written in very detailed and specific way. 1.5 hours were needed to finish the current survey with 140 learning objectives; an additional specific description of the learning objectives would have made the survey too lengthy and reduced the return rate.

The learning objectives of the current study include only cognitive and psychomotor skills. The affective domain was not included. The affective domain includes values, attitudes, behaviors or student motivation for learning, describing how we interact with others [25,26], how we act in the society, how veterinarians care for patients or pet owners, communicate with pet owners and how they demonstrate their morality in particular situations, which is also defined in Good Medical Practice [27].

## Conclusions

With the help of this catalog of learning objectives it is possible to modernize and improve the quality of teaching, curriculum development, competency-based training and outcome-based assessment in veterinary neurology in undergraduate studies in Europe. A comprehensive and effective curriculum is a valuable tool and investment in such a curriculum with one-off development and continual correction can result in enormous benefits for undergraduates and lecturers in terms of time, effectiveness and competency.

## Additional file

Additional file 1:
**Learning objectives with mean values and level distribution for undergraduate.**

## Abbreviations

ACGME: Accreditation council for graduate medical education
ACVIM: American college of veterinary Internal medicine
CNS: Central nervous system
EBVS: European board of veterinary specialisation
ECVN: European college of veterinary neurology
ESVN: European society of veterinary neurology
FCE: Fibrocartilaginous embolus
GME: Granulomatous menigoencephalitis
GMP: The good medical practice
IVDD: Intervertebral disc diesease
PNS: Peripheral nervous system
SRMA: Steroid-reponsive meningitis-arteritis
VetCEE: Veterinary continuous education in europe
WSAVA: World small animal veterinary association

## References

[1] Bloom BS (1984). Taxonomy of educational objectives: The Classification of Educational Goals. Handbook 1: Cognitive Domain.

[2] Conklin J (2005). A taxonomy for learning, teaching, and assessing: a revision of bloom’ s taxonomy of educational objectives. Educ Horiz.

[3] Carroll RG (2001). Design and evaluation of a national set of learning objectives: the medical physiology learning objectives project. Adv Physiol Educ.

[4] Boeker M. Balzer, F, Schulz S. Konzeption einer Ontologie medizinischer Lernziele/Design of an Ontology of Medical Educational Objectives. In: 14. Workshop der gmds-Arbeitsgruppe “Computerunterstützte Lehr- und Lernsysteme in der Medizin (CBT)”und des GMA-Ausschusses “Neue Medien”. 2010; Witten, Germany. doi:10.3205/10cbt35

[5] University of New Mexico School of Medicine. Effective Use Of Performance Objectives For Learning and Assessment; 2005. http://ccoe.umdnj.edu/forms/EffectiveUseofLearningObjectives.pdf, Accessed December 02, 2012

[6] Bloch R, Bürgi H: The Swiss catalogue of learning objectives. Med Teach. 2002. http://www.ncbi.nlm.nih.gov/pubmed/12098433, Accessed January 15, 2013 10.1080/01421590220120759 12098433

[7] Pontes C, EFNS (2001). Task force on postgraduate neurological training survey of the current situation of postgraduate neurological training in Europe. Eur J Neurol.

[8] Charles PD, Scherokman B, Jozefowicz RF (1999). How much neurology should a medical student learn? A position statement of the AAN Undergraduate Education Subcommittee. Acad Med.

[9] Caplan LR, Adelman L (1994). Neurologic education. West J.

[10] Gelb DJ, Gunderson CH, Henry KA, Kirshner HS, Józefowicz RF (2002). The neurology clerkship core curriculum. Neurology.

[11] Platt S, Garosi L. Small Animal Neurological Emergencies. 1st ed. Manson; 2012.

[12] Platt S, Natasha O. editors: BSAVA Manual of Canine and Feline Neurology. 3rd ed. John Wiley & Sons; 2004.

[13] O'Neill DG, Church DB, McGreevy PD, Thomson PC, Brodbelt DC. Longevity and mortality of owned dogs in England. Vet J. 2013;198(3):638-43. 10.1016/j.tvjl.2013.09.020 24206631

[14] Anderson LW, Krathwohl DR (2001). A Taxonomy for Learning, Teaching, and Assessing: A Revision of Bloom’s Taxonomy of Educational Objectives.

[15] Forehand M (2010). Bloom’s Taxonomy - Emerging Perspectives on Learning. Teaching and Technology.

[16] European Association of Establishment for Veterinary Education. The Association: Foundation, Mission and Objectives. http://www.eaeve.org/about-eaeve/history-and-aims.html. Accessed Feburary 20, 2013

[17] Miller GE (1990). The assessment of clinical skills/competence/performance. Acad Med J Assoc Am Med Coll.

[18] Schwartz RW, Donnelly MB, Young B, Nash PP, Witte FM, Griffen WO (1992). Undergraduate surgical education for the twenty-first century. Ann Surg.

[19] Scalese RJ, Issenberg SB (2005). Effective use of simulations for the teaching and acquisition of veterinary professional and clinical skills. J Vet Med Educ.

[20] Waterman E, Hartmann N, Hardy-Cox D, Macleod M, Porr C, Rohr L, Trenholm SB. Interdisciplinary Cooperation in Teaching and Learning at Memorial University. NL, Canada; 2011. http://www.delts.mun.ca/faculty/teachinglearning/ACR_Intdisc_Coop_Report.pdf. Accessed December 20, 2012

[21] Koch M, Fischer MR, Vandevelde M, Tipold A, Ehlers JP (2010). Erfahrungen aus entwicklung und einsatz eines interdisziplinären blended-learning-wahlpflicht- fachs an zwei tiermedizinischen hochschulen einleitung. Zeitschrift Hochschulentwicklung.

[22] Börchers M, Tipold A, Pfarrer C, Fischer MR, Ehlers JP (2010). Akzeptanz von fallbasiertem, interaktivem elearning in der tiermedizin am beispiel des casus-systems. Tierärztliche Praxis Kleintiere.

[23] Pakpoor J, Handel AE, Disanto G, Davenport RJ, Giovannoni G, Ramagopalan SV (2014). National survey of UK medical students on the perception of neurology. BMC Medical Education.

[24] Ridsdale L, Massey R, Clark L (2007). Preventing neurophobia in medical students, and so future doctors. Pract Neurol.

[25] Shephard K (2008). Higher education for sustainability: seeking affective learning outcomes. Int J Sustain High Educ.

[26] Beard C, Clegg S, Smith K (2007). Acknowledging the affective in higher education. Br Educ Res J.

[27] General Medical Council. Good medical practice. 2009. http://www.gmc-uk.org/guidance. Accessed November 22, 2012

