# Supplementary material for: Development of learning objectives for neurology in a veterinary curriculum: part I: undergraduates
Source: BMC Vet Res. 2015 Jan 13;11:2. doi: 10.1186/s12917-014-0315-3 (PMC4300725; doi:10.1186/s12917-014-0315-3)
Supplement: Additional file 1: — Learning objectives with mean values and level distribution for undergraduate. [file 12917_2014_315_MOESM1_ESM.docx]

**Development of learning objectives for neurology in a veterinary curriculum.
Part I: Undergraduates**

**Additional file 1**Learning objectives with mean values and level distribution for undergraduate.

|  | **Level** | | | |
| --- | --- | --- | --- | --- |
| **Learning Objectives1** | **N** | **B** | **A** | **E** |
| N = Not Necessary (blue) (1 - 1.44) |  |  |  |  |
| B = Beginner Level (green) (1.45 - 2.44) |  |  |  |  |
| A = Advanced Level (orange) (2.45-3.44) |  |  |  |  |
| E = Expert Level (red) (3.45 - 4) |  |  |  |  |
| **Anatomy and Physiology** |  |  |  |  |
| 1. Understand the gross neuroanatomic structures of the cat and dog brain and spinal cord |  | 2.19 |  |  |
| 2. Understand the microscopic anatomy of the nervous system |  | 1.69 |  |  |
| 3. Understand the functional neuroanatomy of the central nervous system |  | 2.15 |  |  |
| 4. Understand the functional neuroanatomy of the peripheral nervous system |  | 2.12 |  |  |
| 5. Understand the functional neuroanatomy of the autonomic nervous system |  | 1.99 |  |  |
| 6. Understand the basic principles of neurophysiology in regards to membrane potentials, action potential generation, ion channel conductance and synaptic neurotransmission in the central and peripheral nervous system |  | 1.92 |  |  |
| 7. Understand the principles of cerebrospinal fluid dynamics and intracranial pressure |  | 1.97 |  |  |
|  |  |  |  |  |
| **Pharmacology and Toxicology** |  |  |  |  |
| **> pharmacodynamic and Pharmacokinetic** |  |  |  |  |
| 1. Understand the autonomic nervous system receptors and neurotransmitters |  | 1.84 |  |  |
| 2. Understand the major neurotransmitters and their receptors of the central and peripheral nervous system |  | 1.85 |  |  |
| 3. Understand the mechanisms of drug- delivery through the blood-brain barrier |  | 1.88 |  |  |
| 4. Understand the basic principles of drug absorption, metabolism and clearance |  | 2.07 |  |  |
| 5. Understand the difference between pharmacokinetics and pharmacodynamic qualities of drugs and the parameters used to quantify these qualities |  | 1.78 |  |  |
| 6. Understand therapeutic index in relation to drug efficacy and safety |  | 1.97 |  |  |
| **> Pain** |  |  |  |  |
| 7. Understand the principles of pain activation pathways |  | 2.1 |  |  |
| 8. Understand the mechanism of action of pain therapy |  | 2.19 |  |  |
| **> Neurotoxin** |  |  |  |  |
| 9. Understand the major classes of neurotoxins and the effect on the nervous system |  | 2.03 |  |  |
| **> Antiepileptic drugs** |  |  |  |  |
| 10. Understand the mechanism of the major classes of anti-epileptic drugs |  | 2.07 |  |  |
| 11. Understand the pharmacokinetics of anti- epileptic drugs |  | 1.99 |  |  |
| 12. Understand the side-effect profiles of anti-epileptic drugs |  | 2.21 |  |  |
| **> Immunosuppression** |  |  |  |  |
| 13. Understand the mechanism of immunosuppressive drugs for CNS inflammatory disease |  | 1.94 |  |  |
| 14. Understand the pharmacokinetics of immunosuppressive drugs for CNS inflammatory disease |  | 1.78 |  |  |
| 15. Understand the side-effect profiles of the immunosuppressive drugs for CNS inflammatory disease |  | 2.13 |  |  |
| **> Chemotherapeutic drugs** |  |  |  |  |
| 16. Understand the mechanism of chemotherapeutic drugs for nervous system neoplasia / inflammation |  | 1.75 |  |  |
| 17. Understand the pharmacokinetics of chemotherapeutic drugs for nervous system neoplasia / Inflammation |  | 1.65 |  |  |
| 18. Understand the side-effects profiles of chemotherapeutic drugs for nervous system neoplasia / inflammation |  | 1.88 |  |  |
|  |  |  |  |  |
| **Genetics and Molecular Biology** |  |  |  |  |
| 1. Understand the structure of DNA and a gene |  | 2.08 |  |  |
| 2. Understand the difference between transcription versus translation |  | 2.08 |  |  |
| 3. Understand the genome organization and chromosome structure |  | 2.01 |  |  |
| 4. Understand the inheritance patterns and types of mutations |  | 1.96 |  |  |
| 5. Understand the principles of common molecular genetic tools (laboratory methods; SNPs, microsatellite mapping, candidate genes) |  | 1.65 |  |  |
| 6. Understand the principles of errors of cellular metabolism |  | 1.79 |  |  |
| 7. Understand how to investigate a breed related disorder for an underlying genetic mutation |  | 1.67 |  |  |
|  |  |  |  |  |
| **Clinical Methodology** |  |  |  |  |
| **> Neurologic Examination** |  |  |  |  |
| 1. Perform a neurologic examination of all species |  | 2.24 |  |  |
| 2. Neurolocalize a lesion based on the examination findings |  | 2.37 |  |  |
| **> Laboratory** |  |  |  |  |
| 3. Interpret hematological, serum chemistry and urinalysis results |  |  | 2.58 |  |
| 4. Understand organ function tests (liver, endocrine) |  |  | 2.58 |  |
| 5. Interpret organ function tests (liver, endocrine) |  |  | 2.56 |  |
| **> CSF** |  |  |  |  |
| 6. Understand the risk factors and contraindications of CSF collection and methods to ameliorate these risks. |  | 2.16 |  |  |
| 7. Perform cistern magna collection of CSF in the dog and cat | 1.44 |  |  |  |
| 8. Perform lumbar collection of CSF in the dog and cat |  | 1.47 |  |  |
| 9. Perform lumbar CSF collection in the horse / ruminant / food animal | 1.4 |  |  |  |
| 10. Interpret laboratory results of CSF |  | 1.87 |  |  |
| **> EEG** |  |  |  |  |
| 11. Perform EEG testing in the dog and cat | 1.18 |  |  |  |
| 12. Interpret EEG testing in the dog and cat | 1.21 |  |  |  |
| **> EMG** |  |  |  |  |
| 13. Perform EMG and nerve conduction testing in the dog and cat | 1.23 |  |  |  |
| 14. Interpret EMG and nerve conduction testing in the dog and cat. | 1.35 |  |  |  |
| 15. Perform F-waves, Repetitive stimulation and H-wave testing in the dog and cat. | 1.16 |  |  |  |
| 16. Interpret F-waves, Repetitive stimulation and H-wave testing in the dog and cat. | 1.25 |  |  |  |
| 17. Perform EMG and nerve conduction testing in the horse. | 1.18 |  |  |  |
| 18. Interpret EMG and nerve conduction testing in the horse. | 1.28 |  |  |  |
| 19. Perform EMG and nerve conduction testing in the ruminant / food animal . | 1.17 |  |  |  |
| 20. Interpret EMG and nerve conduction testing in the ruminant / food animal | 1.25 |  |  |  |
| 21. Perform single fiber EMG testing in the dog and cat. | 1.14 |  |  |  |
| 22. Interpret single fiber EMG testing in the dog and cat. | 1.2 |  |  |  |
| **> SSEP (SOMATOSENSORY EVOKED POTENTIAL TESTING)** |  |  |  |  |
| 23. Perform somatosensory evoked potential testing in the dog and cat | 1.14 |  |  |  |
| 24. Interpret somatosensory evoked potential testing in the dog and cat | 1.18 |  |  |  |
| **> BAER (AUDIOTORY EVOKED POTENTIAL TESTING)** |  |  |  |  |
| 25. Perform brainstem auditory evoked potential testing in the dog and cat | 1.26 |  |  |  |
| 26. Interpret brainstem auditory evoked potential testing in the dog and cat | 1.39 |  |  |  |
| **> OEA (OTOACOUSTIC EMISSION TESTING)** |  |  |  |  |
| 27. Perform otoacoustic emission testing in the dog and cat | 1.1 |  |  |  |
| 28. Interpret otoacoustic emission testing in the dog and cat | 1.17 |  |  |  |
| **> VEP (VISUAL EVOKED POTENTIAL TESTING)** |  |  |  |  |
| 29. Perform visual evoked potential testing in the dog and cat | 1.09 |  |  |  |
| 30. Interpret visual evoked potential testing in the dog and cat | 1.15 |  |  |  |
| **> URINARY TRACT ELECTRO. TESTING** |  |  |  |  |
| 31. Perform urinary tract electrodiagnostic testing in the dog and cat | 1.08 |  |  |  |
| 32. Interpret urinary tract electrodiagnostic testing in the dog and cat | 1.14 |  |  |  |
| **> OPHTAMOLOGIC ELECTRO. TESTING** |  |  |  |  |
| 33. Perform ophthalmologic electrodiagnostic testing (ERG, VEP) in the dog and cat. | 1.14 |  |  |  |
| 34. Interpret ophthalmologic electrodiagnostic testing (ERG, VEP) in the dog and cat | 1.25 |  |  |  |
| **> BONE MARROW ASPIRATION & CORE BIOPSY** |  |  |  |  |
| 35. Perform a bone marrow aspirate and or core biopsy |  | 1.54 |  |  |
| 36. Interpret results of bone marrow aspirate and or core biopsy |  | 1.57 |  |  |
|  |  |  |  |  |
| **Disease Mechanisms** |  |  |  |  |
| **> CNS** |  |  |  |  |
| 1. Understand CNS diseases according to the VITAMIN-D principal |  | 2.37 |  |  |
| * Please list the 3 most important diseases of CNS that a undergraduate should know. |  |  |  |  |
| **> PNS** |  |  |  |  |
| 2. Understand PNS diseases according to the VITAMIN-D principal |  | 2.13 |  |  |
| * Please list the 3 most important diseases of PNS that a undergraduate should know. |  |  |  |  |
| **> Seizure** |  |  |  |  |
| 3. Understand the pathogenesis of seizure disorders in dogs and cats |  | 2.12 |  |  |
| 3.1 Understand the diagnosis and treatment of seizure disorders in dogs and cats |  | 2.29 |  |  |
| 4. Understand the pathogenesis of seizure disorders in horses |  | 1.8 |  |  |
| 4.1 Understand the diagnosis and treatment of seizure disorders in horses |  | 1.93 |  |  |
| 5. Understand the pathogenesis of seizure disorders in ruminants / food animals |  | 1.68 |  |  |
| 5.1 Understand the diagnosis and treatment of seizure disorders in ruminants / food animals |  | 1.76 |  |  |
| **> Disc Disease** |  |  |  |  |
| 6. Understand the pathogenesis of disc diseases in dogs and cats |  | 2.32 |  |  |
| 6.1 Understand the diagnosis and treatment of disc diseases in dogs and cats |  | 2.35 |  |  |
| 7. Understand the pathogenesis of disc diseases in horses |  | 1.74 |  |  |
| 7.1 Understand the diagnosis and treatment of disc diseases in horses |  | 1.74 |  |  |
| **> Micturition Disorders** |  |  |  |  |
| 8. Understand the pathogenesis of micturition disorders in dogs and cats |  | 2.09 |  |  |
| 8.1 Understand the diagnosis and treatment of micturition disorders in dogs and cats |  | 2.11 |  |  |
| 9. Understand the pathogenesis of micturition disorders in horses |  | 1.68 |  |  |
| 9.1 Understand the diagnosis and treatment of micturition disorders of horses |  | 1.71 |  |  |
| 10. Understand the pathogenesis of micturition disorders in ruminants / food animals |  | 1.61 |  |  |
| 10.1 Understand the diagnosis and treatment of micturition disorders in ruminants / food animals |  | 1.63 |  |  |
|  |  |  |  |  |
| **Neuroanaesthesia & Neurosurgery** |  |  |  |  |
| **> Theory** |  |  |  |  |
| 1. Understand anesthesia of the neurological patient |  | 1.93 |  |  |
| 2. Understand fluid therapy for a neurological patient |  | 2.1 |  |  |
| 3. Understand peri-operative antibiotic recommendations |  | 2.17 |  |  |
| 4. Understand tissue handling theory and techniques (Biopsies) |  | 1.9 |  |  |
| **> Practical** |  |  |  |  |
| 5. Perform ventral slot |  | 1.53 |  |  |
| 6. Perform thoracolumbar hemilaminectomy |  | 1.61 |  |  |
| 7. Perform dorsal laminectomy of cervical spine |  | 1.49 |  |  |
| 8. Perform fenestration |  | 1.51 |  |  |
| 9. Perform cervical vertebral distraction- fusion | 1.44 |  |  |  |
| 10. Perform ventriculo-peritoneal shunt | 1.44 |  |  |  |
| 11. Perform craniotomy / craniectomy | 1.43 |  |  |  |
| 12. Perform brain biopsy | 1.41 |  |  |  |
| 13. Perform fracture repair |  | 1.54 |  |  |
| 14. Perform dorsal laminectomy of lumbosacral spine |  | 1.52 |  |  |
| 15. Perform atlantoaxial subluxation fixation techniques |  | 1.48 |  |  |
| 16. Perform muscle biopsy |  | 1.66 |  |  |
| 17. Perform nerve biopsy |  | 1.54 |  |  |
|  |  |  |  |  |
| **Neuroradiology** |  |  |  |  |
| **> Theory** |  |  |  |  |
| 1. Understand CT scanning technique |  | 1.8 |  |  |
| 2. Understand CT physics |  | 1.66 |  |  |
| 3. Understand MRI scanning technique |  | 1.71 |  |  |
| 4. Understand MRI physics |  | 1.57 |  |  |
| 5. Understand nervous system ultrasound technique |  | 1.5 |  |  |
| 6. Understand nuclear medicine technique | 1.44 |  |  |  |
| 7. Understand radiation therapy principles |  | 1.54 |  |  |
| **> Practical** |  |  |  |  |
| 8. Interpret radiographs of the abdomen and thorax |  |  | 2.5 |  |
| 9. Interpret radiographs of the axial and appendicular skeleton |  | 2.41 |  |  |
| 10. Interpret radiographs of the skull |  | 2.18 |  |  |
| 11. Interpret CT scan of the brain and skull |  | 1.73 |  |  |
| 12. Interpret CT scan of the vertebral column and spinal cord |  | 1.73 |  |  |
| 13. Interpret MRI scans of the brain |  | 1.71 |  |  |
| 14. Interpret MRI scans of the spine |  | 1.7 |  |  |
| 15. Interpret MRI scans of the peripheral nervous system |  | 1.59 |  |  |
| 16. Identify the different MRI scan techniques and their use in clinical practice |  | 1.59 |  |  |
| 17. Iinterpret myelograms in the cat, dog and horse |  | 1.71 |  |  |
| 18. Perform myelography in the dog and cat | 1.42 |  |  |  |
| 19. Perform myelography in the horse | 1.29 |  |  |  |
| 20. Perform nervous system ultrasound interpretation | 1.35 |  |  |  |
| 21. Perform nuclear medicine interpretation | 1.29 |  |  |  |
| 22. Apply radiation therapy technique | 1.27 |  |  |  |
|  |  |  |  |  |
| **Pathology** |  |  |  |  |
| 1. Understand hematological cytological interpretation |  | 2.25 |  |  |
| 2. Understand the technique to acquire CSF samples in small animals |  | 1.92 |  |  |
| 3. Understand the technique to acquire CSF samples in large animals |  | 1.75 |  |  |
| 4. Exhibit competence in CSF cytological interpretation in small animals |  | 1.61 |  |  |
| 5. Exhibit competence in CSF cytological interpretation in horses/ruminants/food animals |  | 1.51 |  |  |
| 6. Exhibit competence in CSF sample examination (protein content, cell counting) |  | 1.54 |  |  |
| 7. Understand basic CNS pathological interpretation |  | 1.7 |  |  |
| 8. Understand basic PNS pathological interpretation |  | 1.65 |  |  |
| 9. Understand microscopic pathological features of specific small animal diseases |  | 1.63 |  |  |
| 10. Understand microscopic pathological features of specific horse diseases |  | 1.54 |  |  |
| 11. Understand microscopic pathological features of specific ruminant / food animal disease |  | 1.53 |  |  |
| 12. Understand Infectious disease testing techniques (PCR / Western blot / Serology) |  | 1.87 |  |  |
| 13. Understand Infectious disease testing interpretation |  | 1.96 |  |  |
| 14. Exhibit competence in bone marrow cytological interpretation |  | 1.61 |  |  |
| 15. Exhibit competence in brain biopsy cytological interpretation | 1.42 |  |  |  |
|  |  |  |  |  |
|  |  |  |  |  |

* Free text questions
